# Propofol vs Midazolam As the Initial Sedation Strategy for Mechanically Ventilated Patients: A Single-Center Experience From Saudi Arabia

**DOI:** 10.7759/cureus.66090

**Published:** 2024-08-03

**Authors:** Ali S Al-Shareef, Kholoud Babkair, Jamil M Baljoon, Tiaf A Alkhamisi, Areen Altwairqi, Hassan Bogari, Bsaim Altirkistani, Najd Alsukhayri, Majed Ramadan

**Affiliations:** 1 Department of Emergency Medicine, Ministry of National Guard-Health Affairs, Jeddah, SAU; 2 Research Department, King Abdullah International Medical Research Center, Jeddah, SAU; 3 Department of Medicine, College of Medicine, King Saud bin Abdulaziz University for Health Sciences, Jeddah, SAU; 4 Department of Research, King Abdullah International Medical Research Center, Jeddah, SAU; 5 Department of Surgery, King Abdullah International Medical Research Center, Jeddah, SAU; 6 College of Medicine, King Saud bin Abdulaziz University for Health Sciences, Jeddah, SAU; 7 Department of Biomedical Research, King Abdullah International Medical Research Center, Jeddah, SAU

**Keywords:** length of stay in icu, invasive mechanical ventilation, propofol, midazolam, 28-day mortality

## Abstract

Background

Propofol and midazolam are the most common sedative agents used in critical settings. Propofol and midazolam might have different mortality rates after sedation administration. Some studies mention that propofol is associated with a lower mortality rate than midazolam in mechanically ventilated patients, but other studies have contradicting results. This study aims to compare the 28-day mortality of propofol versus midazolam for patients undergoing mechanical ventilation in the National Guard Hospital Health Affairs (NGHA)-Western Region (WR).

Methods

A retrospective chart review was conducted at (NGHA-WR) from March 2016 to July 2022. The inclusion criteria were those mechanically ventilated patients aged 18 years or older who were admitted to ICU, where they were given either propofol or midazolam as the initial sedative agent. Those who signed DNR (Do Not Resuscitate) or were contraindicated to sedation, such as allergy, were excluded from the study. Data were retrospectively retrieved and obtained from the Hospital Information System (HIS-BestCare, Saudi-Korean Health Informatics Company, Riyadh, Saudi Arabia) and the Office of Data Intelligence.

Results

There is a significant difference between the type of sedation and the 28-day mortality rate. Midazolam was associated with higher rates of mortality - 104 (47.93%) when compared to propofol - three (14.29%). Also, patients who used midazolam had longer durations of ICU stay compared to propofol, with a mean number of 19.23 days vs 7.55 days, respectively.

Conclusion

There is a significant difference regarding the 28-day mortality between patients who were given propofol or midazolam as an initial sedative agent for mechanical ventilation ≥ 24 hours. Moreover, the use of propofol is associated with fewer days of being intubated or being in ICU when compared to midazolam.

## Introduction

Continuous intravenous infusion sedation is commonly used in the mechanical ventilation (MV) setting [1]. In contrast to intermittent bolus infusion, continuous intravenous sedation is expected in the Intensive Care Unit (ICU) since it provides more levels of both sedation and comfort [2-3]. Propofol and midazolam are the most common sedative agents used in critical settings [4-5]. However, other less frequently used sedatives include ketamine, ketafol, etomidate, dexmedetomidine, and methohexital [6-7]. Propofol is considered a potent sedative, hypnotic, and anxiolytic drug without a significant analgesic effect. Also, one of the properties of propofol is that it rapidly crosses the blood-brain barrier, leading to a fast onset and short recovery time [8-9]. Propofol's onset of action is less than 1 minute, with the duration of action having clinical effects for 10 minutes [10]. Furthermore, propofol has a short weaning time since the initial half-life is approximately 40 minutes, and the terminal half-life is between 4 to 7 hours [10]. This might be explained due to the fact that propofol is excreted by both the liver and kidneys [10]

On the other hand, midazolam is a common sedative benzodiazepine agent that is used for ICU sedation [11]. It has a “ceiling effect” because it only binds to Gamma-aminobutyric acid (GABA) receptors. Additionally, due to the large volume distribution of midazolam, it can cause accumulation during continuous infusion [12]. Some benzodiazepines such as midazolam have a shorter elimination half-life since they are eliminated from the body in 1.5 to 2.5 hours [13]

Regarding safety profile, there are some differences between propofol and midazolam. One example is the drop in blood pressure after administrating the sedation. Propofol decreases mean blood pressure, systolic blood pressure, and diastolic blood pressure more than midazolam [14]. 

Furthermore, using midazolam with an opioid is associated with respiratory depression and cardiac arrest [15]. Propofol and midazolam might have different mortality rates after sedation administration in critically ill patients. One study showed that propofol has a lower mortality rate in mechanically ventilated patients at 19.7% compared to 28.8% in midazolam [16]. This statement is also confirmed by a meta-analysis and systematic review that included 23 studies, which concluded that propofol is associated with better outcomes and less ICU stay when compared to midazolam [17]. However, another study states that patients who had propofol have a higher mortality rate than those who had fentanyl and/or midazolam, with a mortality rate of 28% vs 3% in catheter-directed thrombolysis patients [18]. There are certain studies discussing the effects of using propofol vs midazolam as sedative agents on mortality in mechanically ventilated patients in ICU, as aforementioned. Yet, there is a lack of studies addressing its effects on general intensive care patients at our institution in Saudi Arabia. Therefore, this study aimed to compare the 28-day mortality of propofol versus midazolam for patients undergoing mechanical ventilation in the ICU in National Guard Hospital Health Affairs, Jeddah, Saudi Arabia.

## Materials and methods

Study design and settings

A retrospective cohort chart review study was conducted at National Guard Health Affairs (NGHA), a tertiary hospital, in King Abdulaziz Medical City in Jeddah, Saudi Arabia (KAMC-J) from May 2016 to July 2022.

Identification of study participants

The inclusion criteria encompassed mechanically ventilated patients aged 18 years or older, admitted to the intensive care unit, and were administered either propofol or midazolam as a sedative agent, either alone or in combination with lorazepam, for a duration exceeding 24 hours. The study exclusively considered patients falling within the specified timeframe from May 2016 to July 2022. Conversely, patients who had signed a Do Not Resuscitate (DNR) order, those who had received both propofol and midazolam concurrently during the same treatment, individuals who had undergone intubation on more than one occasion, and those who had been administered midazolam or propofol in conjunction with ketamine, ketafol, etomidate, or dexmedetomidine were excluded from the study. The sampling technique employed a non-random consecutive sampling approach, including all 233 patients who met the predetermined inclusion and exclusion criteria in this study.

Study measures, covariates, and outcome variables

Data for the study was retrieved from the hospital's electronic records using the BestCare 2.0 System (Saudi-Korean Health Informatics Company, Riyadh, Saudi Arabia). A data collection sheet containing various study variables was employed to collect the required information. Our primary outcome was the Initial Sedation Strategy (propofol vs midazolam). Other potential confounding variables encompassed demographic and clinical data as covariate factors, which were retrospectively obtained. The demographic characteristics recorded included the patient's gender, age, reason for admission, and any comorbidities present. Additionally, clinical data encompassed variables such as 28-day mortality, source and reason for admission, type of medication administered, use of lorazepam, duration of intubation, length of stay in the intensive care unit (ICU), duration of hospitalization, systolic and diastolic blood pressure readings, heart rate, respiratory rate, oxygen saturation levels, presence of severe head trauma, and the occurrence of severe spinal trauma. All collected data were meticulously entered into an Excel (Microsoft Corporation, Redmond, USA) file by the research team and maintained in strict confidentiality throughout the study.

Data analysis

Our analytical approach commenced with a univariate analysis aimed at examining the clinical and demographic characteristics of the study participants, categorized by the type of sedative administered, namely, propofol vs. midazolam. For categorical variables, we employed the Chi-square test, while the Fisher exact test was applied to variables with fewer than 5 subjects. Numeric variables were assessed using the t-test. In the subsequent multivariate analysis, we harnessed a binary multiple logistic regression model, incorporating the exact option to accommodate the study's relatively small sample size effectively. During this analysis, we rigorously assessed the multicollinearity assumption and found no evidence of high correlations among the independent variables. Data analysis was conducted using Macintosh Project JMP Pro 15.2.0 (JMP Statistical Discovery, Cary, USA) and SPSS Statistics 28.0.1.1 (IBM Corp., Armonk, USA), ensuring robust and comprehensive statistical evaluations (14). Statistically significant results were identified by p-values less than 0.05.

## Results

ICU patient characteristics at NGHA

A total of 233 patients were included in this study, out of which 126 (54.08%) were male. The mean age was 48 (95% CI = 44.08 - 51.93), and most of the included patients were admitted from the emergency department, with a total of 129 (55.37%). Regarding the reason for admission, 31 (13.3%) patients had more than one cause of admission. Moreover, respiratory causes were the most common reason for admission, which constituted 101 (43.35%) patients, while septic shock was the second reason for admission seen in 17 (7.3%) patients.

Out of 233 patients, 212 (90.99%) were sedated by the administration of midazolam, while 21 (9.01%) patients were sedated by propofol. Overall, of the 233 patients, 22 (49.4%) were also sedated by Lorazepam. The mean duration of mechanical ventilation was 13.98 days (95% CI = 6.89 - 21.08). The mean of the length of stay in the ICU and length of hospitalization was 18.48 (95% CI = 10.96 - 25.98) days, respectively.

Regarding the vital signs in the first hour after administrating the sedative agent, the mean systolic pressure of the included patients was 107.97 mmHg (95% CI = 103.5 - 112), while the mean diastolic pressure was 59.7 mmHg (95% CI = 57.39 - 62.02). Moreover, the mean heart and respiratory rates were 106.13 beats per minute (95% CI = 102.14 - 110.13) and 28.22 breaths per minute (95% CI = 26.29 - 30.14), respectively.

Predictors of the outcome

**Figure 1:**
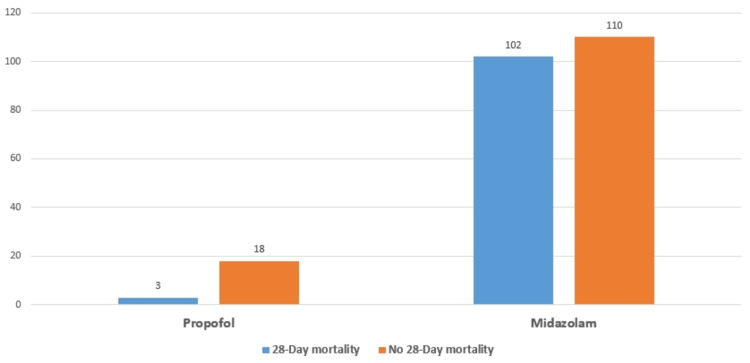
Comparison of the 28-day mortality between the use of midazolam and propofol

Regarding the 28-day mortality, there was a significant difference between using midazolam and propofol for patients who were on mechanical ventilation (MV). Midazolam was associated with higher rates of 28-day mortality since the 28-day mortality was 102 (48.11%) in midazolam compared to propofol, which was three (14.29%), with p = 0.0031, as illustrated in Figure 1.

In addition, there is a significant difference between midazolam and propofol regarding the duration of MV and the length of ICU stay. The mean duration of MV in the midazolam group was 14.81 days (95% CI = 7.37 to 22.26), which is higher than the propofol group, which had a mean duration of 5.62 days (95% CI = -17.99 to 29.23). Also, patients who were administered midazolam had a longer duration of ICU stay compared to propofol, with 19.51 days (95% CI = 11.66 to 27.36) and 7.55 days (95% CI= -18.02 to 33.12), respectively, with p = 0.026.

There was a significant difference regarding some of the vital signs that were documented in the first hour of sedation administration between patients who were on midazolam and patients who were on propofol during MV. The mean systolic blood pressure was lower in patients who used midazolam compared to propofol, 105.66 mmHg (95% CI = 101.25 to 110.07) and 129.05 mmHg (95% CI = 116.68 to 141.42), respectively, with p = 0.001. Also, the mean of RR was higher in patients who used midazolam - 29.18 breaths per minute (95% CI = 27.12 to 31.16) compared to propofol - 18.71 breaths per minute (95% CI = 12.5 to 24.93) (Table 1).

**Table 1:** A comparison of the length of ICU stay and vital signs of the study patients during the first hour after the sedation administration between midazolam and propofol. *P-value represents the significance of Mann-Whitney U test ^†^P-value represents the significance of t-test

Parameter	Midazolam	Propofol	P-value
Length of ICU stay (95% CI) N = 233*	19.51 days (11.66 – 27.36)	7.55 days (-18.02 – 33.12)	0.026
Systolic blood pressure (95% CI) N = 233†	105.66 (101.3 – 110.1)	129.05 (115.2 – 142.8)	0.001
Diastolic blood pressure (95% CI) N = 233*	59.1 (56.7 – 61.5)	65.6 (57.9 – 73.3)	0.053
Heart Rate (95% CI) N = 233*	107.3 (103.1– 111.5)	94.4 (81.18 – 107.7)	0.097
Respiratory Rate (95% CI) N = 228*	29.18 (27.2– 31.2)	18.71 (12.5 – 24.9)	<0.001
Oxygen saturation (95% CI) N = 231*	94 (92.7– 95.2)	96.5 (92.6 – 100.46)	0.232

There was no significant association between the sedation type and the following factors: gender of the patient, age of the patient, admission source, the reason for admission, length of hospitalization, diastolic BP, heart rate (HR), Oxygen saturation, comorbidities of the patient, and head or spinal injury (Table 2).

**Table 2:** The demographic characteristics of the study patients *P-value represents the significance of Chi-Square ^†^P-value represents the significance of Mann-Whitney U test

Parameter	Midazolam	Propofol	P-value
Gender (%)*	N = 212 (100%)	N = 21 (100%)	0.279
Male	117 (55.2%)	9 (42.9%)	
Female	95 (44.8%)	12 (57.1%)	
Age (95% CI) †	47.2 (43.1 – 51.4)	55.7 (42.6 – 68.8)	0.489
Source of admission (%)*	N = 212 (100%)	N = 21 (100%)	0.289
Emergency department	114 (53.8%)	15 (71.4%)	
Transferred from another hospital	15 (7%)	0 (0%)	
Labor and Delivery department	14 (6.6%)	0 (0%)	
Surgery department	4 (1.9%)	1 (4.8%)	
Other sources	65 (30.7%)	5 (23.8%)	
Reason of admission (%)*	N = 212 (100%)	N = 21 (100%)	0.504
Respiratory	70 (33%)	5 (23.8%)	
Septic shock	38 (17.9%)	2 (9.5%)	
Neurological	18 (8.5%)	3 (14.3%)	
Surgical	17 (8.0%)	3 (14.3%)	
Trauma	9 (4.3%)	1 (4.8%)	
Cardiovascular	7 (3.3%)	3 (14.3%)	
Gastrointestinal	4 (1.9%)	0 (0%)	
Other single causes	14 (6.6%)	1 (4.8%)	
Multiple causes	35 (15.5%)	3 (14.3%)	
Was there a severe head injury?(%) *	N = 212 (100%)	N = 21 (100%)	0.526
Yes	4 (1.9%)	0 (0%)	
No	208 (98.1%)	21 (100%)	
Was there a severe spinal injury? (%) *	N = 212 (100%)	N = 21 (100%)	N/A
Yes	0 (0%)	0 (0%)	
No	212 (100%)	21 (100%)	
Length of hospitalization (95% CI) †	34.7 (25.6 – 43.9)	19.2 (-9.8 – 48.2)	0.637

In the binary logistic regression model, the dependent variable was the type of sedation. The independent variables were gender, age of the patient, 28-day mortality, admission source, whether there were additional sedative drugs or not, whether there was head injury or not, length of ICU stay, length of hospitalization, duration of MV, systolic BP, diastolic BP, HR, and oxygen saturation. The only significant variable between midazolam and propofol in the binary logistic regression model was the 28-day mortality, which illustrated that patients who used midazolam had an adjusted odds ratio of 28-day mortality of 8.98 when compared to the usage of propofol, with p = 0.003 (Table 3). 

**Table 3:** Binary logistic regression analysis between the midazolam group and the propofol group

Factor	Adjusted odds ratio	P-value
Age	0.49	0.486
Gender	.017	0.897
28-Day Mortality	8.98	0.003
Admission source	3.33	0.068
Additional sedatives	0.288	0.592
Was there head injury	0.00	0.999
The duration of mechanical ventilation	0.07	0.799
Length of ICU stay	0.14	0.710
Length of hospitalization	0.65	0.420
Systolic BP	0.97	0.324
Diastolic BP	0.03	0.873
Heart rate	0.23	0.634
Oxygen saturation	0.98	0.322
Constant	0.000	0.999

## Discussion

In this study, we investigated the 28-day mortality of both midazolam and propofol, and we found that midazolam had a higher 28-day mortality compared to propofol, with approximate numbers of 48% and 14%, respectively. In addition, patients who used midazolam had an adjusted odds ratio of 8.98 of having a 28-day mortality than patients who used propofol. Our results are in line with the results of Garcia R et al., who did a meta-analysis and systematic review that included 23 studies to compare propofol and midazolam and concluded that propofol is associated with better outcomes [17]. Also, Sun W et al. found that patients who use midazolam have a higher 28-day mortality rate of 30.8%. In contrast, patients who used propofol had a 28-day mortality rate of 25.5% [19]. Also, Sun W et al. concluded that patients who use midazolam have an adjusted odds ratio of 1.42 of having more than 28 days mortality when compared to patients who use propofol [19]. In addition, the aforementioned results are supported by similar results concluded by Lonardo NW et al., who claim that mechanically ventilated patients who were on propofol had less mortality in comparison to patients who were on midazolam [16]. These results might be explained by the fact that propofol has a fast onset, fast weaning time, and fast recovery time [8-10].

In contrast to these results, Manchec B et al. state that in patients who are diagnosed with acute submissive pulmonary embolism and underwent catheter-directed thrombolysis, out of the 25 patients who were given propofol, seven (28%) had mortality [14]. However, in the 111 patients who were given midazolam/fentanyl, the mortality was only in three patients (3%) [18]. This contrariety could be explained by the fact that we tested the 28-day mortality specifically, while Manchec B et al. tested mortality with no time frame, or the fact that propofol is associated with a significant decrease in mean blood pressure, systolic blood pressure, and diastolic blood pressure than midazolam [14].

Regarding the ICU stay factor, we also found that the length of ICU stay was higher in the midazolam group than in the propofol group, with 19.5 days and 7.6 days, respectively. These results of the ICU stay were similar to what Garcia R et al., mention in their systematic review [17]. Also, patients who used midazolam had a higher mean duration on mechanical ventilation of 14.8 days when compared to patients on propofol who had a mean of 5.6 days. It can be concluded that the usage of propofol in patients who undergo mechanical ventilation for more than 24 hours is associated with fewer days of being intubated or being in ICU when compared to midazolam. Therefore, it can be beneficial for the patient to be sedated by propofol instead of midazolam since prolonged MV is associated with adverse outcomes, and the likelihood of developing adverse outcomes of MV is decreased when the period of being intubated is reduced [20-21].

In this study, the vital signs were measured during the first hour of administering the sedative agent. The systolic blood pressure was calculated to be higher in patients who were given propofol when compared to patients who were given midazolam for sedation - a mean of 129 mmHg and 105.7 mmHg, respectively. Our data show that usually, the baseline parameters of the patients who undergo MV are more hemodynamically stable during the first hour of propofol sedation. This might be explained by the fact that physicians usually avoid giving propofol as a sedative agent to patients who are hemodynamically unstable since propofol decreases blood pressure more than midazolam, as mentioned in Xu AY et al. who state that propofol decreases blood pressure by 39.26 mmHg and midazolam decreases the systolic blood pressure by 25.8 mmHg after the first hour of sedation administration [22]. Also, if a patient was prescribed propofol and then was switched to midazolam due to being hemodynamically unstable, the patient was excluded from our study, as aforementioned. Regarding the respiratory rate (RR) during the first hour of sedation administration, patients who were given propofol had a lower rate of RR when compared to midazolam, with 18.7 and 29.2 breaths per minute, respectively. This is in contrast to Xu AY et al. who mentioned that there is no significant difference between the RR in patients who were given propofol or midazolam as sedation [22].

Limitations

This study still has some inherent limitations of retrospective research. Despite excluding some confounding factors, some of our inclusion factors might interfere with the propofol and midazolam effect, which is caused by the study design being a retrospective. Also, this study was conducted in a single center, which may result in both a small sample size and a low representation of patients. Moreover, even though the investigators chose a consecutive sampling technique, the number of patients who were sedated by propofol was much lower than the number of patients who were on midazolam. This might be explained by the availability of propofol or propofol being a relatively new drug. 

Therefore, it is recommended that prospective studies be conducted for more accurate results. Also, the authors recommend conducting multicenter studies to compare the data of the centers. Unlike other studies, our primary endpoint was comparing the 28-day mortality of using propofol and midazolam, while previous studies compared additional drugs like etomidate and ketamine. Therefore, this study lacks the assessment of other sedative drugs.

## Conclusions

There is a significant difference in the 28-day mortality of patients who were given propofol vs midazolam as a sedative agent for mechanical ventilation ≥ 24 hours in our ICU. Moreover, the use of propofol is associated with fewer days of ICU stay and fewer days of being intubated when compared to midazolam. Future prospective and or randomized controlled trials are recommended to solidify this conclusion and change practices.
